# Editorial: The Role of Flower Color in Angiosperm Evolution

**DOI:** 10.3389/fpls.2021.736998

**Published:** 2021-09-17

**Authors:** Eduardo Narbona, Montserrat Arista, Justen B. Whittall, Maria Gabriela Gutierrez Camargo, Mani Shrestha

**Affiliations:** ^1^Department of Molecular Biology and Biochemical Engineering, Pablo de Olavide University, Seville, Spain; ^2^Department of Plant Biology and Ecology, Faculty of Biology, University of Seville, Seville, Spain; ^3^Department of Biology, College of Arts and Sciences, Santa Clara University, Santa Clara, CA, United States; ^4^Laboratory of Ecology and Evolution of Plant-Animal Interactions, Institute of Biosciences, São Paulo State University, Botucatu, Brazil; ^5^Disturbance Ecology, Bayreuth Center for Ecology and Environmental Research, University of Bayreuth, Bayreuth, Germany; ^6^School of Media and Communication, Royal Melbourne Institute of Technology University, Melbourne, VIC, Australia; ^7^Faculty of Information Technology, Monash University, Melbourne, VIC, Australia

**Keywords:** flower color diversity, anthocyanins, flower color evolution, pollination, selective pressure

Although angiosperms exhibit a wide range of variability in floral traits such as shape and size, flower color is a hallmark of angiosperm diversity. Since before Darwin's time, flower color has long been appreciated for its role in pollinator attraction (Sprengel, 1793; Mendel, 1866; Darwin, 1895; Faegri and van der Pijl, 1966; Proctor and Yeo, 1973). However, over the past few decades, a growing body of evidence suggests that flower color can be molded by a diversity of selective pressures. The rapid accumulation of flower color studies has spurred several thorough reviews (Winkel-Shirley, 2001; Koes et al., 2005; Rausher, 2008; Sobel and Streisfeld, 2013; Narbona et al., 2018; Sapir et al., 2021), but here we present the largest collection of investigations specifically focused on the role of flower color in angiosperm evolution.

This Research Topic is composed of 28 studies on the role of flower color in angiosperm evolution. These contributions include species living on nearly all continents plucked from most major branches of the angiosperm tree of life (Figure 1). Investigations span traditional scales in biology, from gene expression and biochemical profiles to pollinator perception and community assembly. Evolutionarily, studies range from within species flower color polymorphisms to macroevolutionary patterns of flower color evolution within and among genera. Ecologically, investigations span a diversity of plant communities including neotropical savannas, temperate serpentine seeps, subtropical mountains, and tropical dry forests.

**Figure 1:**
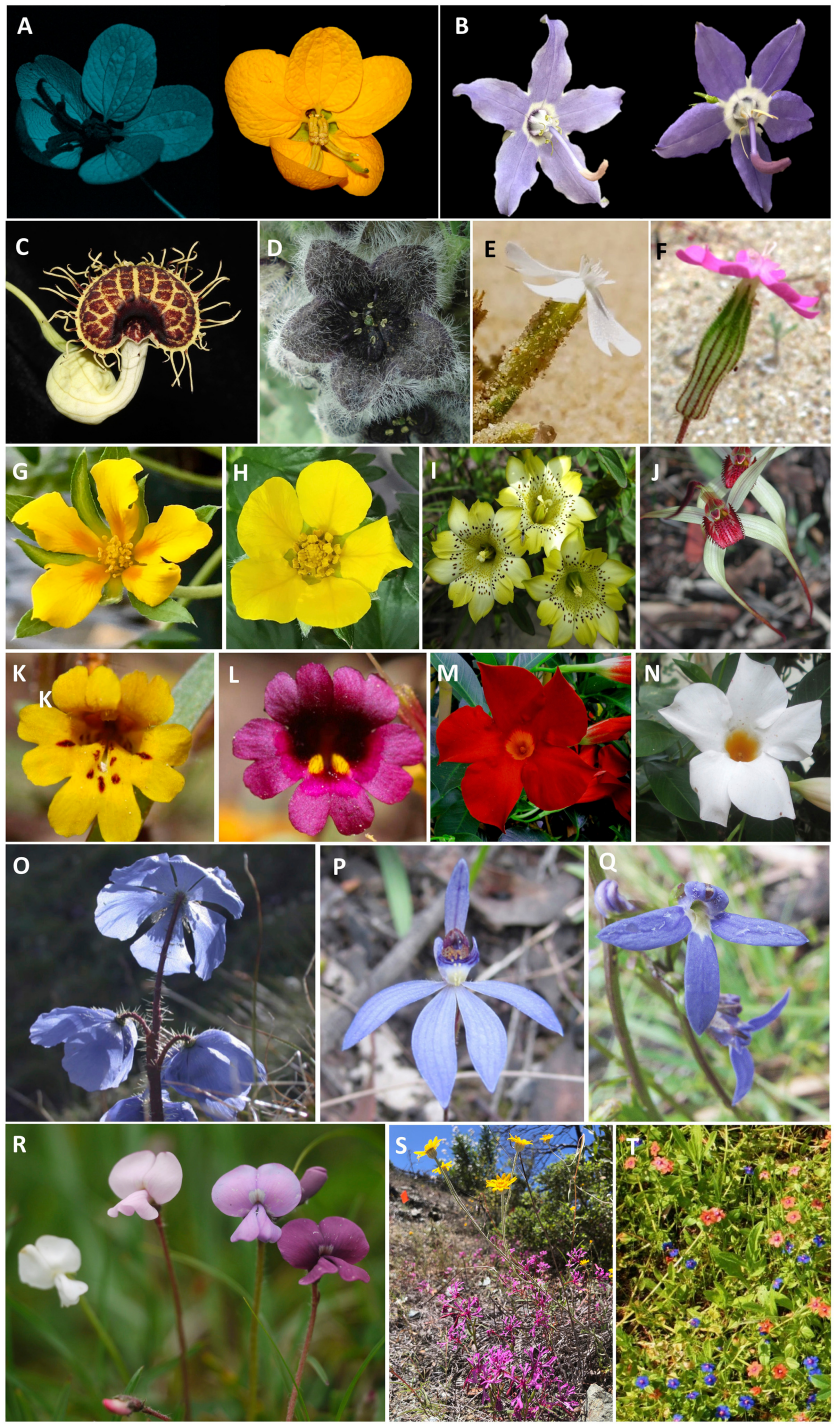
Examples of species included in our Research Topic “*The Role of Flower Color in Angiosperm Evolution*.” **(A)** *Senna rugosa* (Fabaceae) using UV (left) and conventional photography (right) (Brazil, Mariah di Stasi). **(B)** Flower color variation in *Campanula americana* (Campanulaceae, USA, Matthew Koski). **(C)** *Aristolochia fimbriata* (Aristolochiaceae, Colombia, Natalia Pabón-Mora). **(D)** *Jaborosa rotacea* (Gesneriaceae, Argentina, Marcela Moré). **(E,F)** White and pink flowers of *Silene littorea* plants (Caryophyllaceae, Spain, Eduardo Narbona). **(G,H)** *Potentilla plattensis* and *Argentina anserine* (Rosaceae, USA, Matthew Koski). **(I)** *Gentiana flavomaculata* (Gentianaceae, Taiwan, Chun-Neng Wang). **(J)** *Caladenia fulva* (Orchidadeae, Australia, Ann Lawrie). **(K,L)** Flower color morphs of *Erythranthe discolor* (Phrymaceae, USA, Naomi Fraga and Dena Grossenbacher). **(M,N)** Flower color morphs of *Mandevilla sanderi* (Apocynaceae, The Netherlands, Doekele Stavenga). **(O)** *Meconopsis horridula* (Papaveraceae, China, Anke Jenstsch). **(P)** *Caladenia caerula* (Orchidaceae, Australia, Mani Shrestha). **(Q)** *Lobelia rhombifolia* (Campanulaceae, Australia, Mani Shrestha). **(R)** Flower color variation in *Tibetia yunnanensis* (Leguminosae, China, Klaus Lunau). **(S)** Plants of *Eriophyllum lanatum* (Asteraceae) and *Clarkia concinna* (Onagraceae) coexisting (USA, Gerardo Arceo-Gómez). **(T)** Flower color morphs of *Lysimachia arvensis* coexisting (Primulaceae, Spain, Montserrat Arista).

Fundamentally, flower color depends on the underlying pigments and recent studies have begun to decipher the genetic basis of this pigment production. The most prevalent and variable pigments in flowers are the anthocyanins, which originate from the flavonoid biosynthetic pathway (Tanaka et al., 2008). Branches of this pathway produce other flavonoid compounds (e.g., flavonols, flavones, isoflavones, proanthocyanins, and catechins) that protect plants against a variety of environmental stressors such as pathogens, herbivores, drought, extreme temperatures, ultraviolet (UV) radiation, etc. (Pollastri and Tattini, 2011; Falcone Ferreyra et al., 2012; Jiang et al., 2016). Thus, certain floral colors not only affect pollinator attraction (or avoidance), but may also determine resistance to biotic and abiotic stresses (Strauss and Whittall, 2006; Landi et al., 2015).

## The Genetics and Biochemistry of Anthocyanin Production

The anthocyanin biosynthetic pathway is mostly regulated at the transcriptional level (Albert et al., 2014). In this volume, Muñoz-Gómez et al. studied the regulatory gene evolution in the anthocyanin biosynthetic pathway among the Aristolochiaceae, a family with a high floral diversity exhibiting elaborate color patterns. The authors conclude that the anthocyanin biosynthetic pathway and its regulatory genes are largely conserved across the family, and color variation is primarily determined by differences in gene expression. Stavenga et al. studied the pH dependence of flavonoid absorption spectra of two Papaveraceae species, *Papaver dubium* (red) and *Meconopsis cambrica* (orange), and white and red varieties of *Mandevilla sanderi*. The authors found that the absorption spectrum (i.e. colour) of anthocyanins can be dependent on the pH of the vacuole, however, not all flavonoids respond similarly to changes in vacuolar pH. Color diversity is also remarkably high in Gesnerioideae and Ogutchen et al. have elegantly shown that this is due to the expanded use of the anthocyanin biosynthetic pathway. In this family, the anthocyanin biosynthetic pathway includes the deoxyanthocyanin branch, which is rarely functional in angiosperms. Ogutchen et al. also call for a better understanding of the link between the biochemical basis of flower color and the visual perception of the primary pollinators. At the microevolutionary scale, Sánchez-Cabrera et al. identified several anthocyanin biosynthetic pathway loci likely involved in the shift in flower color in the orange/blue polymorphic *Lysimachia arvensis*. Further, these authors found differential expression of two genes (*F3*′*5*′*H* and *DFR*) in the anthocyanin biosynthetic pathway. The biochemical analysis of color morphs was consistent with the transcriptome data indicating that the shift from blue to orange petals is caused by a change from primarily malvidin to largely pelargonidin forms of anthocyanins (Sánchez-Cabrera et al.). Authors discuss that both the decreased expression of *F3'5'H* in orange petals and the differential expression of two distinct copies of *DFR*, which also exhibit amino acid changes in the color-determining substrate specificity region, strongly correlate with the blue to orange transition. Collectively, these studies have revealed the complexity and nuance of the pigment biochemistry, yet the consistent role of gene expression changes in the anthocyanin biosynthetic pathway underlying the diversity of flower colors.

## How Is Color Perceived?

Flower color is in the eye of the beholder. Color perception depends on the sensory abilities of different groups of pollinators (van der Kooi et al., 2021). Flower color variation can arise when pollinators with diverse sensory systems drive fitness differences between flower color variants (Koski, 2020). Thus, in order to understand the selective role of pollinators in flower color, it is necessary to incorporate their color perception. Garcia et al. shows that experienced pollinators often make correct decisions about the presence of rewards based on flower color, but this is not the case for inexperienced pollinators. These authors concluded that color cannot be considered an inherent trait because its interpretation by an animal's brain is frequently context-dependent.

Although most studies of color perception and foraging decisions have been traditionally based on *Apis mellifera*'s sensory system (e.g., Giurfa et al., 1994; Dyer et al., 2008; Rohde et al., 2013), we are in dire need of experimental studies on the sensory capabilities of non-*Apis* bee pollinators. In this volume, Koethe et al. have carried out a comparative study of food source selection between two stingless bee species and honeybees. These three species reacted similarly to color, but the variation among them could be the result of adaptations to the bees' respective habitat and morphological constraints. Thus, habitat traits can influence color perception by pollinators. In a similar conceptual framework, Martins et al. studied how seasonal changes in the leaf-background colouration of Brazilian savanna communities affect the perception of flower color contrasts by bees. They found that background coloration affected flower contrasts, favoring flower conspicuousness to bees according to the season and providing new insights regarding the temporal patterns of plant–pollinator interactions.

Dyer et al. reviewed the old concept of the rarity of blue flowers. They found that short wavelength reflecting blue flowers are indeed frequent in nature when considering the color vision of bees and they point out that competition for pollinators may drive the evolution of blue flowers. Coimbra et al. used the visual system of bees to test the generalization of the bee-avoidance hypothesis proposed to explain why bird pollinated flowers tend to be red. Their results suggest that bee sensory exclusion *via* color signals is exclusive to bird flowers, while non-bee, insect flowers might use other sensory channels to exclude bees. In another study, Whitney et al. analyzed how flower color variation within plant populations of bee- and hummingbird-pollinated plant species is perceived by their specific pollinators. They found that bees sensed equal color variation within species from the two pollination systems, but birds perceived more color variation in bird-pollinated flowers than in bee-pollinated flowers.

## Flower Color Variation: A Broad Scale Approach

Phylogenetically controlled studies at the community level have repeatedly found that flower color tends to show weak phylogenetic signal, reflecting an underlying pattern of evolutionary divergence (e.g., McEwen and Vamosi, 2010; Muchhala et al., 2014; Ortiz et al., 2021). For example, Tai et al. studied flower color signaling in Taiwan and found that although high altitude floras tend to be phylogenetically clustered, their flower colors exhibited only weak phylogenetic signal. Thus, they suggest that flower color signaling was mainly influenced by color preferences of key bee pollinators. Most studies on the phylogenetic signal of flower color focus on the predominant color exhibited by the flowers, although in many species flowers are not uniform showing contrasting colors (i.e., color patterns) that are perceived by pollinators (Hempel de Ibarra et al., 2015). Some color patterns include reflection of UV light that is perceived by pollinators that have UV photoreceptors (van der Kooi et al., 2021). When floral color patterns in the ultraviolet spectrum (UV patterns) are investigated, contrasting results have been found. Tunes et al. studied patterns of floral UV reflectance in plants from a Neotropical savanna. They tested the roles of phylogenetic relatedness and pollinator mediated selection on the distribution of UV floral patterns. They confirmed that phylogenetic relatedness constrains the diversity of floral UV patterns, however, the distribution of floral UV-features could not be ascribed to a single ecological or evolutionary factor. This study calls for a deeper understanding of ecological and evolutionary processes involved when interpreting floral UV color diversification. Some flowers of the Potentilleae tribe (Rosaceae) have petals with UV patterns, whereas others show human-visible patterns or uniform petal color. Koski investigated the evolutionary transition between patterned and non-patterned petals and found that the presence of UV and human-visible patterns evolved independently from one another. He also found that the evolution of human-visible patterns was associated with the evolution of larger flowers, supporting the hypothesis that nectar or pollen guides are more likely to evolve in larger-flowered species. In another study, Roguz et al. explored the evolution of flower color in *Iris*, a genus displaying a huge diversity of flower color and color patterns among and within species. They found that the most recent common ancestor likely had entomophilous, monochromatic flowers.

The fate of new color variants depends on a diversity of evolutionary forces. Flower color can affect pollinator attraction and, when investigated in a community context, flower color can mediate different types of interactions among co-flowering species, such as competition, facilitation or mimicry (Ghazoul, 2006; Johnson and Schiestl, 2016; Kemp et al., 2019). The abundance and species richness of the local habitat may also influence the type or strength of ecological interactions among species. Co-flowering species with similar flower colors may compete for pollinator services; selection could thus favor differentiation to improve pollinator recognition and fidelity (Gumbert et al., 1999; McEwen and Vamosi, 2010). Competition for pollinators would result in a pattern of phylogenetic over-dispersion of floral color among co-flowering species (Sargent and Ackerly, 2008). Alternatively, similarity in flower color may result from selection for standardization of the signals that improves recognition by pollinators and increases their visitation rates. LeCroy et al. studied 14 serpentine seep communities in California differing in species richness and size. In smaller-sized communities they found that competitive exclusion could be a dominant process shaping lower species richness, but this process is less detectable in larger, more speciose communities. Similarly, Moré et al. explored the potential importance of pollinators as drivers of floral color diversification in the genus *Jaborosa* taking into account the perceptual abilities of their pollinators (i.e., nocturnal hawkmoths versus diurnal saprophilous flies) with a geographical perspective. This study found that the ability of plants that colonized the newly formed environments during Andean orogeny and the ecological changes that followed were concomitant with transitions in flower color as perceived by different pollinator functional groups. Further, this study suggests that habitat and pollination are linked to the history of a plant's lineage.

## Flower Color Variation at the Microevolutionary Scale

From a microevolutionary point of view, intraspecific variation in floral color is widely distributed (Narbona et al., 2018). Flower color may vary continuously or discretely, the latter situation being much rarer. Evolutionary mechanisms explaining the maintenance of intraspecific flower color variation include both biotic and abiotic factors, although other processes can also play an important role (Narbona et al., 2018). Trunschke et al. reviewed studies addressing selection on continuous flower color variation in the context of pollinator interactions. They suggest that evidence for significant pollinator-mediated selection is surprisingly limited among existing studies. In fact, most of the current understanding of flower color evolution arises from variation between discrete color morphs, where selection by pollinators is usually one of the most important factors involved in color variation. Selective foraging by pollinators for specific color morphs is frequently reported (Ortiz et al., 2015). These studies focus on the most common pollinators such as bees or birds, while selection by other groups is rarely reported.

Two studies in this volume have investigated microevolutionary flower color variation in South-African species pollinated by less-commonly-reported pollinator groups. Ellis et al. studied patterns of color distribution of discrete white and orange daisy species pollinated by bombyliids. They found that the dominant pollinator in orange communities has strong preferences for orange flowers while the dominant pollinator in white communities exhibited an innate preference for white flowers. These findings demonstrate that landscape-level flower color turnover is likely shaped by a strong qualitative geographic mosaic of bee-fly pollinators with divergent color preferences. Similarly, Johnson et al. found that patterns of color variation in *Drosera cistiflora*, a species pollinated by beetles, are associated with different pollinator communities. Given that these beetle species discriminate among color forms (von Witt et al., 2020), the authors conclude that beetle pollinators are a significant factor in the evolution of *D. cistiflora* flower color. This is one of the first reports of flower color selection by beetles (see Streinzer et al., 2019).

Although direct selection on flower color by pollinators has been widely explored, indirect selection on correlated traits is rarely reported (e.g., Gómez, 2000). Other floral traits can be correlated with flower color and thus, to understand the role of pollinators as selective agents on flower color it is necessary to consider such associations. The number of flowers per plant, a component of floral display, is one of the most important traits affecting pollinator attraction, and its association with flower color has been studied in *Silene littorea* (Rodríguez-Castañeda et al.) and *Medicago sativa* (Brunet et al.). Both studies show significant correlations between flower color and floral display and demonstrate significant directional selection on floral display that indirectly selects for flower color. These examples of correlational selection can partly explain flower color evolution in *M. sativa* and the maintenance of the flower color polymorphism in *S. littorea*.

The synthesis of floral pigments can also have pleiotropic effects on defensive plant compounds and consequently herbivores can play a role in flower color variation (Strauss and Whittall, 2006). Specifically, anthocyanin pigments in floral tissues can influence herbivore preference and performance (Irwin et al., 2003; Frey, 2004). In this volume, Sobral et al. found evidence of transgenerational effects of herbivory on flower color variation in *Raphanus sativus*. Epigenetic modifications due to herbivory influences the proportion of plants with anthocyanins in the following generation showing a link between biotic ecological interactions across generations and plasticity in flower color; with some exceptions this phenomenon is virtually undescribed in natural plant populations.

Abiotic factors also play an important role in flower color variation (Strauss and Whittall, 2006). Water availability, temperature and solar radiation can select flowers with higher pigment concentrations either directly or indirectly, giving rise to geographical patterns of flower color variation (Dalrymple et al., 2020). The role of abiotic factors on flower color variation is more frequently reported and, in this volume, it has been addressed in two monkeyflowers– *Erythranthe discolor* and *Diplacus mephyticus* (Grossenbacher et al.), *Campanula americana* (Koski and Galloway), and *Drosera cistifolia* (Johnson et al.). Drought stress is one of the most important factors predicting flower color across geographic ranges (Warren and Mackenzie, 2001; Arista et al., 2013). Grossenbacher et al. found a higher frequency of anthocyanin producing morphs in populations with reduced precipitation for two monkeyflower species as a consequence of the protective role of anthocyanins. However, after accounting for phylogeny, there was no evidence that drought stress leads to a macroevolutionary pattern in flower color across monkeyflowers. In *Campanula americana* local temperature appears to shape the geographical pattern of flower color intensity, although the genetic population structure seems to be driven by historical effects, an important factor rarely considered (Koski and Galloway). Conversely, other rarely studied, potential abiotic selective agents (such as soil type) do not seem to be related to the geographic pattern of color variation in *Drosera cistifolia* (Johnson et al.). The role of abiotic factors on flower color variation has also been reported in this volume. Peach et al. studied the effect of UV radiation on flower color variation in *Clarkia unguiculata* and found that populations growing in areas of high UV radiation showed higher anthocyanin concentration, a pattern previously reported (Del Valle et al., 2015). However, contrary to expectations, UV-absorbing floral patterns did not have a direct “pollen protection” function, highlighting the need for research on a wider range of taxa to understand the role of anthocyanins in flower protection against UV radiation [see Kay et al. (2020)]. These studies addressing the role of abiotic factors affecting flower color variation highlight the protective role of pigments under some conditions of abiotic stress, but also reveal the multifaceted evolutionary and ecological complexity underlying natural flower color variation.

Other less known factors involved in the maintenance of intraspecific flower color variation are also reported in this volume. Jiménez-López et al. explored the role of selfing as a way to maintain the flower color polymorphism in *Lysimachia arvensis*. They found that in Mediterranean populations, both biotic and abiotic factors select against one of the color morphs, but that increased selfing in this morph preserves the color variation within populations. One consequence of this mating system by flower color interaction is a decrease in genetic variation that could have macroevolutionary consequences.

Flower color also shows intrafloral variation giving rise to complex patterns that can attract and guide bee pollinators. Aguiar et al. studied intrafloral color variation in *Cattleya walkeriana*, and found a centripetally increasing spectral purity within the flowers of this bee pollinated orchid species. This intrafloral variation was unrelated to the development of floral structures, suggesting an important role of pollinator selection in the modularization of flower color.

Lastly, the importance of taking flower color into account in conservation strategies is highlighted in this special topic. Orchidaceae is one of the most threatened plant families, as it has very large numbers and proportions of endangered species worldwide. Intraspecific flower color variability in the endangered orchid *Caladonia fulva* has been suggested to be the result of hybridization with *C. reticulata*. Genetic and breeding studies by Basist et al. clearly shows that *C. fulva* is a flower color polymorphic species and conservation of the color variants is essential to maintain genetic diversity.

In this Research Topic, we present a modern synthesis of flower color studies. Clearly, understanding the evolution of flower colors in angiosperms requires a diversity of perspectives across scales, both ecological and evolutionary, from the molecular, biochemical, physiological, anatomical, organismal, population, and community levels. These studies show that flower color in plant populations and plant communities are often the result of a combination of biotic and abiotic selective pressures and are highly context dependent. They clearly indicate the need to incorporate new approaches and innovative methodologies when studying flower color evolution.

## Author Contributions

All authors contributed to the article and approved the submitted version.

## Funding

Research by MA, EN, JW, and MC was supported by the European Regional Development Fund (ERDF) and grants from the Spanish government (PID2020-116222GB-I00) and the Andalusian Regional Ministry of Economy, Knowledge, Business and University (PY18-3651, US-1265280, and UPO-1261687). MC received scholarships from CAPES Coordination of Superior Level Staff Improvement (grant #88887.583309/2020-00 Finance Code 001) and FAPESP São Paulo Research Foundation (grant #2015/10754-8). Research by MS was funded by the German Federal Ministry of Education (BMBF) grant number 031B0516C and by Alan Dorin and Adrian G. Dyer under the funding scheme from the Australian Research Council Discovery Project 160100161.

## Conflict of Interest

The authors declare that the research was conducted in the absence of any commercial or financial relationships that could be construed as a potential conflict of interest.

## Publisher's Note

All claims expressed in this article are solely those of the authors and do not necessarily represent those of their affiliated organizations, or those of the publisher, the editors and the reviewers. Any product that may be evaluated in this article, or claim that may be made by its manufacturer, is not guaranteed or endorsed by the publisher.
